# Differentiating Variations in Thumb Position From Recordings of the Surface Electromyogram in Adults Performing Static Grips, a Proof of Concept Study

**DOI:** 10.3389/fbioe.2019.00123

**Published:** 2019-05-22

**Authors:** Alejandra Aranceta-Garza, Bernard Arthur Conway

**Affiliations:** Department of Biomedical Engineering, University of Strathclyde, Glasgow, United Kingdom

**Keywords:** grip formation, high-density surface-electromyography, machine learning, prosthetics, self-organizing featured maps, thumb position control, upper-limb myoelectric prosthetics

## Abstract

Hand gesture and grip formations are produced by the muscle synergies arising between extrinsic and intrinsic hand muscles and many functional hand movements involve repositioning of the thumb relative to other digits. In this study we explored whether changes in thumb posture in able-body volunteers can be identified and classified from the modulation of forearm muscle surface-electromyography (sEMG) alone without reference to activity from the intrinsic musculature. In this proof-of-concept study, our goal was to determine if there is scope to develop prosthetic hand control systems that may incorporate myoelectric thumb-position control. Healthy volunteers performed a controlled-isometric grip task with their thumb held in four different opposing-postures. Grip force during task performance was maintained at 30% maximal-voluntary-force and sEMG signals from the forearm were recorded using 2D high-density sEMG (HD-sEMG arrays). Correlations between sEMG amplitude and root-mean squared estimates with variation in thumb-position were investigated using principal-component analysis and self-organizing feature maps. Results demonstrate that forearm muscle sEMG patterns possess classifiable parameters that correlate with variations in static thumb position (accuracy of 88.25 ± 0.5% anterior; 91.25 ± 2.5% posterior musculature of the forearm sites). Of importance, this suggests that in transradial amputees, despite the loss of access to the intrinsic muscles that control thumb action, an acceptable level of control over a thumb component within myoelectric devices may be achievable. Accordingly, further work exploring the potential to provide myoelectric control over the thumb within a prosthetic hand is warranted.

## Introduction

**Figure 1:**
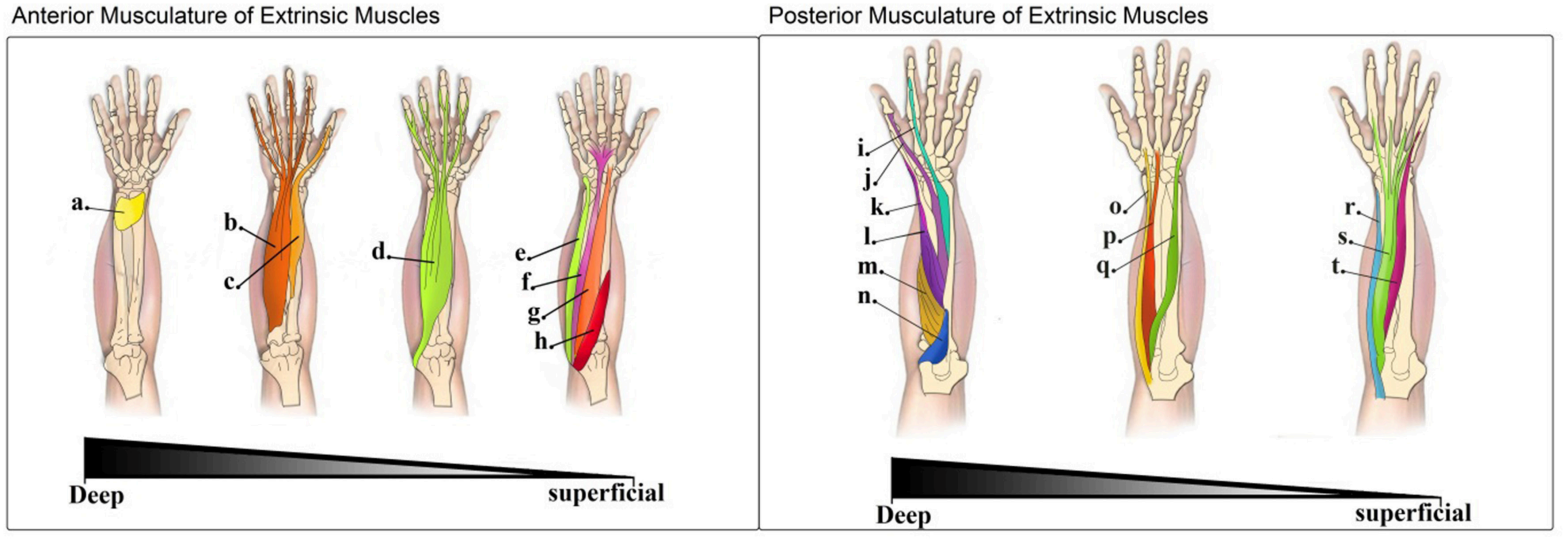
**Left:** Muscles in the anterior compartment of extrinsic muscles (flexor muscles of the forearm)—The muscles of the anterior compartment of the forearm are depicted in this image from the deepest layer (left) to the most superficial one (right): (a). Pronator quadratus (PQ); (b). Flexor digitorium profundus (FDP); (c). Flexor pollicis longus (FPL); (d). Flexor digitorium superficialis (FDS); (e). Flexor carpi ulnaris (FCU); (f). Palmaris longus (PL); (g). Flexor carpi radialis (FCR); (h). Pronator teres (PT). **Right:** Muscles in the posterior compartment of extrinsic muscles (extensor muscles of the forearm)—The muscles of the posterior compartment of the forearm are depicted in this image moving from the deepest to the most superficial layer: (i). Extensor indicis (EI); (j). Extensor pollicis longus (EPL); (k). Extensor pollicis brevis (EPB); (l). Abductor pollicis longus (APL); (m). Supinator (S); (n). Anconeous (A); (o). Extensor carpi radialis longus (ECRl); (p). Extensor carpi radialis brevis (ECRb); (q). Extensor carpi ulnaris (ECU); (r). Brachioradialis (B); (s). Extensor digitorium (ED); (t). Extensor digiti minimi (EDM).

In forming hand gestures and grip patterns, activation of both extrinsic and intrinsic hand muscles is necessary (Maier and Hepp-Reymond, 1995). In all aspects of grip formation, the thumb plays a vital role. The thumb is the digit that displays the highest level of independent-fractionated control and the highest level of functional coupling with other digits during grip tasks (Ingram et al., 2008). Importantly, the intrinsic hand muscles that act on the thumb play a critical role in determining both opposing grip strength and thumb positioning. In healthy subjects, these muscles are aided by the actions of the deep extrinsic hand musculature (Figure 1). However, in transradial and partial-hand amputees this normal interplay between extrinsic and intrinsic muscle synergy is lost. Only the residual forearm muscles within the remaining limb segment are accessible and usable for myoelectric prosthetic control. This creates a challenging control problem, as without direct access to the hand muscles and a lack in specificity in sampling, the potential for intuitive control of prosthetic thumb is significantly compromised.

The surface electromyogram (sEMG) is a small amplitude, time-varying signal detected from the surface of the skin and reflects the summed activity of superficial and deep motor-units from near-by co-contracting muscles. Activity in motor-units is driven by neural commands acting through the motor nerves innervating those muscles. Therefore, sEMG signals are directly linked to the neural-command associated with movement. This makes the use of sEMG a compelling non-invasive method for user control and interfacing in prostheses, and has resulted in a range of commercial myoelectric prosthetic devices entering the market over the last decades. However, the control over grip-transitions during activities of daily living remains a significant challenge for the majority of myoelectric prosthetic users to master. Intention detection based on user-modulation of sEMG, through implementation of real time classification algorithms, adaptive learning methods, binary classifications or pattern recognition (Englehart et al., 2001; Englehart and Hudgins, 2003; Ajiboye and Weir, 2005; Yonghong et al., 2005; Parker et al., 2006; Amsüss et al., 2014; Castro et al., 2015) can support effective object handling and manipulation in expert users of myoelectric prostheses. Yet, despite the potential functional gains these devices can provide to experienced users, control for many users remains a significant challenge and no active user control over thumb position is achievable.

Current control of commercially-available hand prostheses is driven by sampling parameters of sEMG from residual muscles of the amputee's forearm (Ohnishi et al., 2007; Cipriani et al., 2011). Accordingly, in most myoelectric devices the control of thumb flexion across the palm, and opposition to digits is predetermined through pre-set grip selection features. This approach while simple to implement, does not lend itself to intuitive user control.

With advances in mechatronics and robotics the mechanical capabilities of hand prostheses will continue to improve, and powered-thumb mechanisms will become commonplace in multiarticulating hands. With this, there will be an increase in the variety of functional grip gestures and postures available to users. However, without improvements in user-control over grip and gesture formation, the utility of these devices will not be realized and continued user abandonment of high-cost devices will remain problematic for the industry and healthcare providers.

**Figure 2:**
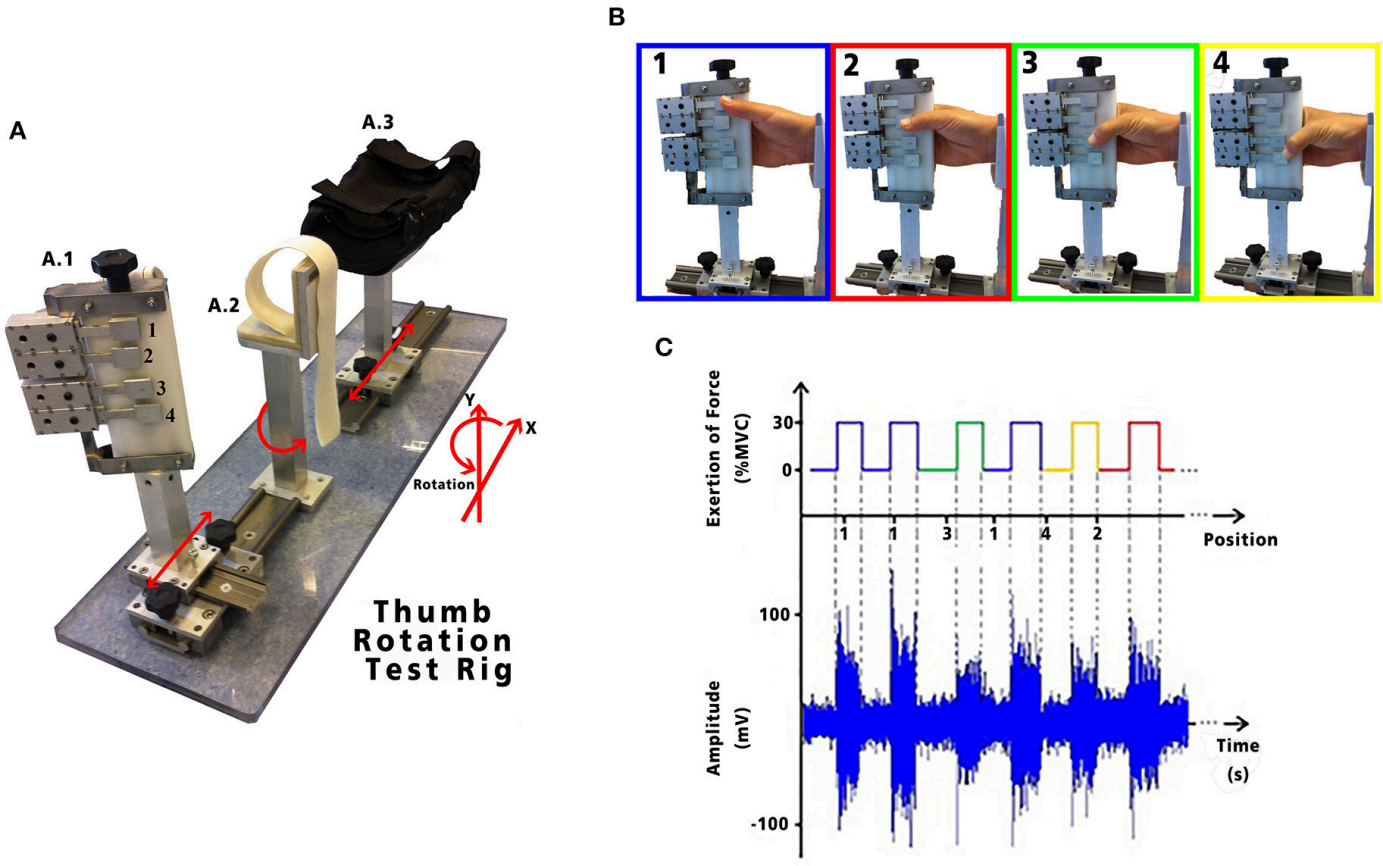
**(A)** Test apparatus used to examine thumb opposition; (A.1) fingers holder: allows freedom of movement in the X and Y axis, as well as rotational movement with four strain gauges represented by the numbers 1, 2, 3, and 4. Each strain gauge corresponds to the 4 digits: secundus digitus manus, digitus medius, digitus annularis, and digitus minimus manus, respectively. Each LSR could be moved in the X and Y axis to allow adjustment to each subject's anatomical measurements; (A.2) wrist holder: fixed padded holder that avoided any flexion/extension of the wrist, minimizing activity in the forearm; and (A.3) elbow holder: with movement in the X axis, to allow adjustment for subject comfort, minimizing muscle contractions caused by pronation, supination, flexion or extension of the forearm. HD-sEMG data acquired while performing the tasks are shown with its synchronous exertion of force. **(B)** Static thumb positions performed by each volunteer. **(C)** Exemplary sEMG signals from one channel of the HD-sEMG highlighting the different amplitudes for the different static grips.

Control over thumb opposition is critical in providing dexterous hand function and a number of biomechanical studies illustrate that in order to exert effective opposition grip force, there is a fundamental requirement for coactivation across many different muscle groups, not all of which act directly on the thumb (Li and Tang, 2007). This in itself dictates that valuable information on thumb opposition may be recoverable from identifying differences in the EMG activities generated during different thumb opposition tasks. In this proof-of-concept study, we have investigated in able-bodied volunteers whether the patterns of forearm sEMG can be used to differentiate between static grips of equal force formed when the thumb is held in different thumb opposition postures (see Figure 2B). The objective of this study was to determine if different opposition positions of the thumb could be determined from the sampled forearm sEMG signals. Our study focused on forearm sEMG signals because these are the muscle groups likely to be preserved in cases of hand amputation. HDsEMG recordings were used in order to provide a detailed profile of the localized amplitude and activity patterns occurring in forearm muscles during task execution.

## Methods

The aim of this research was to explore and investigate the variations in sEMG patterns recovered from the intact forearm whilst a volunteer performed a controlled grip task with their thumb held at different postures. To this end, a protocol requiring repeatable performance of static isometric grip tasks was implemented and involved sampling HD-sEMG and grip force during contractions performed with the thumb held in opposition to the base of digits 2, 3, 4, and 5, respectively (see Figures 2B,C).

### Research Subjects/Participants

This study was approved by the University of Strathclyde Research Ethics Committee and in accordance with the Declaration of Helsinki recruited seven healthy right-handed participants (6 male, 1 female; age 28.5 ± 3.7 years). All subjects provided informed written consent prior to the tests and reported to have no prior history of nerve damage, hand surgery, existing neuromuscular pain, tremor, epilepsy, and/or movement disorder.

### Experimental Design

Subjects were seated and the position of their upper arm, forearm and wrist was standardized by use of a specially designed apparatus incorporating grip force measurement (Figure 2A). Experiments were performed on the subjects' right arm which was held at 90° of shoulder abduction, 90° of elbow flexion and the wrist in a neutral position. Four adjustable finger pads incorporating strain gauges configured to measure the force exerted between the thumb and hand were aligned in opposition to the subject's 2nd, 3rd, 4th, and 5th metacarpalphalangeal joints. The strain gauged finger pads were calibrated and the resulting force signals amplified and filtered (DC-10 Hz) prior to digitization for use in real-time visual force feedback to the participant. The opposition task we describe here is equivalent to placing the distal phalanx of the thumb in opposition to each of the four dermal papillae lying along the palmer digital crease. Each different grip position reflects a different degree of thumb flexion across the palm but in each case the resultant target grip force was identical.

Once subjects could demonstrate that they could perform a grip that targeted each finger pad, an estimate of the maximal voluntary contraction (MVC) force between the thumb and hand was determined. Graphical user interfaces (GUIs) were then used to provide the participant with visual cues indicating which finger pad to oppose, the start and end times for a contraction effort and a 30% MVC grip force target level.

Simultaneous HD-sEMG signal acquisition occurred whilst the subject followed the GUI instructed grip tasks. The GUI randomized the opposition order of grip tasks to provide a total of 30 grip trials at each of the four grip postures (120 trials in total), and each 30% MVC task was sustained for 5 s. A variable resting time interval of 5–7 s between efforts was provided. No subject reported fatigue nor was fatigue evident as a failure to sustain the required force throughout a session.

### HD-sEMG Recording Setup

Monopolar HD-sEMG signals were acquired using 128 channels of a multichannel bioelectrical signal amplifier (EMG-USB2, OT-Bioelettronica, Italy). Data were acquired from participants performing the different thumb opposition grip tasks as described above.

**Figure 3:**
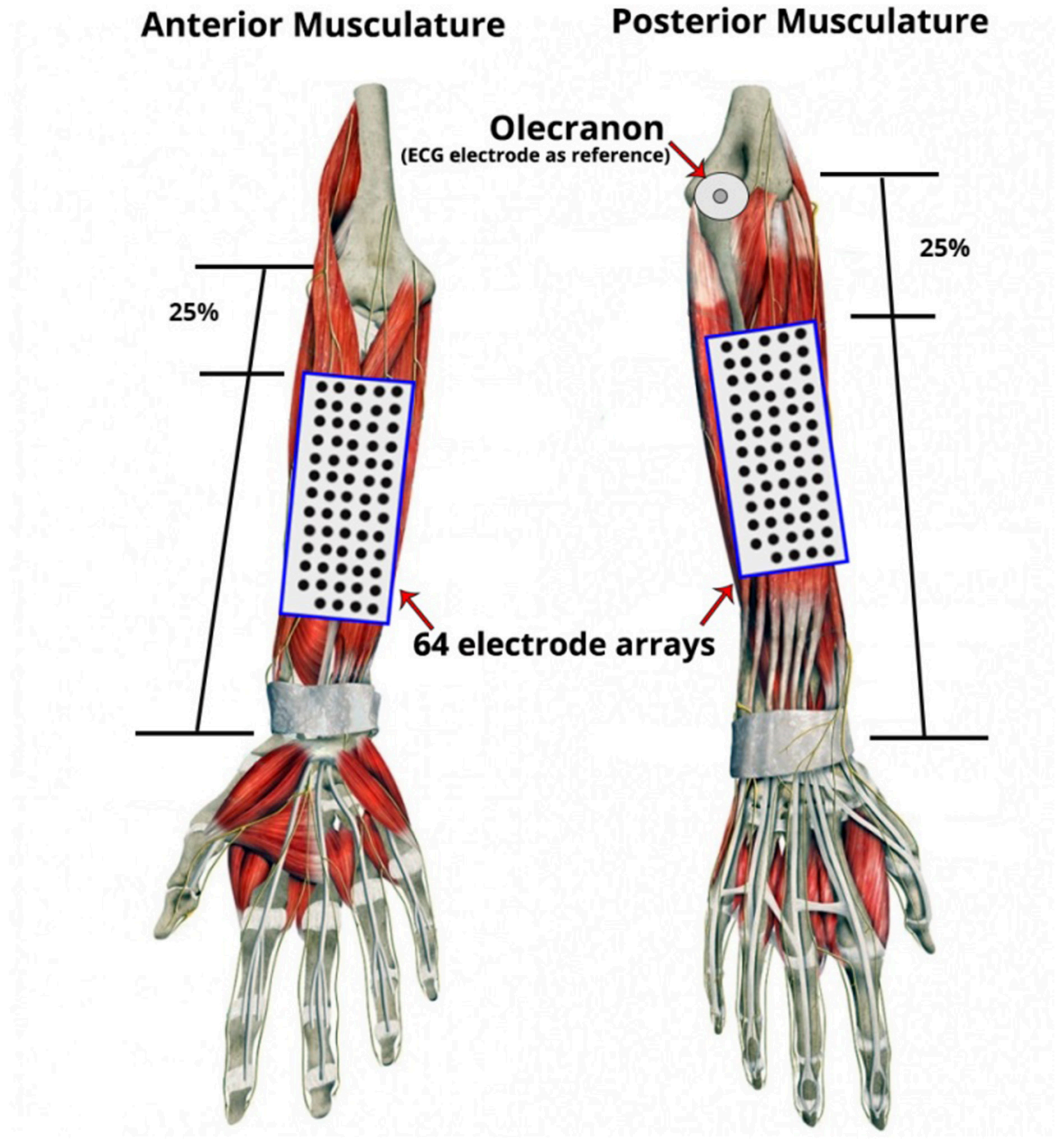
Placement of electrode grids on the regions of interest (ROI): Anterior and Posterior Musculature. Two 13 by 5 electrode grids were placed over the skin of the forearm based on the anatomical landmarks of the forearm and relative to each participant's forearm length and circumference dimensions. To standardize grid positioning across subjects, a simple proportional measurement based on distances between surface anatomical landmarks was employed. The distance between the ulnar head and the olecranon (for the posterior musculature) and the ulnar head to the elbow crease (for the anterior musculature) were measured and the respective grids aligned to a virtual line 25% from the proximal landmarks. An additional disposable ECG electrode was placed on the olecranon as the reference electrode.

Two regions of interest (ROIs) were recorded simultaneously via two separate 13-by-5 electrode grids with an 8 mm inter-electrode distance (ELSCH064R3S, OT-Bioelettronica, Italy). The two ROIs corresponded to sites overlying the posterior and the anterior forearm musculature (Figure 3). To standardize the positioning of the grids across subjects, the distance between the ulnar head and the olecranon (for the posterior musculature) and the ulnar head to the elbow crease (for anterior musculature) were measured and the respective grids aligned to a virtual line 25% from the proximal landmarks. The electrode grids were fixed to the skin using specialized self-adhesive pads (KITAD064, OT-Bioelettronica, Italy) and breathable medical tape. Under the recording conditions experienced in these experiments and due to the use of arm and wrist supports there were few instances of movement artifact within the array recordings.

### Amplitude Signal Analysis

The HD-sEMG signals were acquired at a frequency of 2,048 samples per second, with a fixed gain of 1,000 V/V, a bandwidth of 3–900 Hz and a zero-phase notch filter to remove the 50 Hz mains interference and its harmonics. The sampled HD-sEMG data was then used to create iso-potential maps computed from averaged RMS values for each channel. The averaged RMS values were calculated, as detailed in Sebelius et al. (2006) and Betthauser et al. (2018), for both ROI across windows of 300 with 50 ms overlap.

The RMS is a convenient measure reflecting the change of electrical activity generated by a contracting muscle and is widely used for feature recognition/distribution (Nielsen et al., 2009).

To be able to observe the presence of four distinguishable thumb oppositions during each set of 30% MVC exertions, feature extraction was applied as an initial investigation to determine if each of these positions were separable. Feature extraction, based on work presented by de Luca et al. (1982) and further explored by Phinyomark et al. (2012), was implemented as a quantitative method to extract the distinguishable information from sEMG parameters (i.e., Integrated-EMG; Mean Absolute Value; RMS). This method was applied using the Matlab Classification Learner toolbox (R2015a) to vectors containing the averaged 300 ms overlapping windows of RMS separated by posture with the resulting distribution visualized using pair-wise scatterplots. These plots were then used to evaluate the properties of each position-feature in space by observation of the resulting clusters.

Thereafter, iso-potential maps were virtually constructed to mirror the electrode arrays positioned on the forearm to aid visualization of the changes in sEMG topography and activation patterns seen during grip task performance. A further global iso-potential map of the averaged RMS windows (grand average) was calculated to highlight the local differences specific to each of the four thumb positions in both ROIs.

As differences in the global RMS values are seen to vary within each thumb posture during different grip tasks, feature extraction was applied to the averaged 300 ms overlapped windows of RMS vectors separated by thumb opposition and by grid, in order to understand the amplitude variation for each task.

Finally, the thumb variations were classified using self-organizing feature maps (SOFM). The SOFM is an unsupervised artificial neural-network algorithm which aims to discover underlying structure by clustering the input data quantified by their Euclidean distance (Hassan et al., 2012). Each HD-sEMG channel was conditioned as a feature vector (FV) which contained the corresponding isometric voluntary contraction (IVC) for all repetitions of the four different thumb grip formations for each ROI as described and developed by Kohonen (1982). This guaranteed that no electrode had a stronger influence over others during the clustering analysis.

### Data Validation

As it is essential to quantify the performance of the SOFM, Levenberg-Marquardt (LM) and the Neural-Network Pattern Recognition (NNPR) Matlab built-in toolboxes were applied as supervised methods. These methods use backpropagation and are designed to minimize the sum of square error [24, 25], thereby providing to be a suitable benchmark. The networks were presented with 30% of the labeled data, 35% used for testing and the remaining 35% was used for validation. The function used to assess the maps performance was the mean squared error value (MSE) between the outputs and the targets, where lower values meant greater accuracy and zero meant no error.

## Results

In each experiment, the participant opposed their thumb to each of the four different target postures, as previously described, totaling 120 repetitions (30 per opposition).

### Amplitude Signal Analysis and Feature Extraction

**Figure 4:**
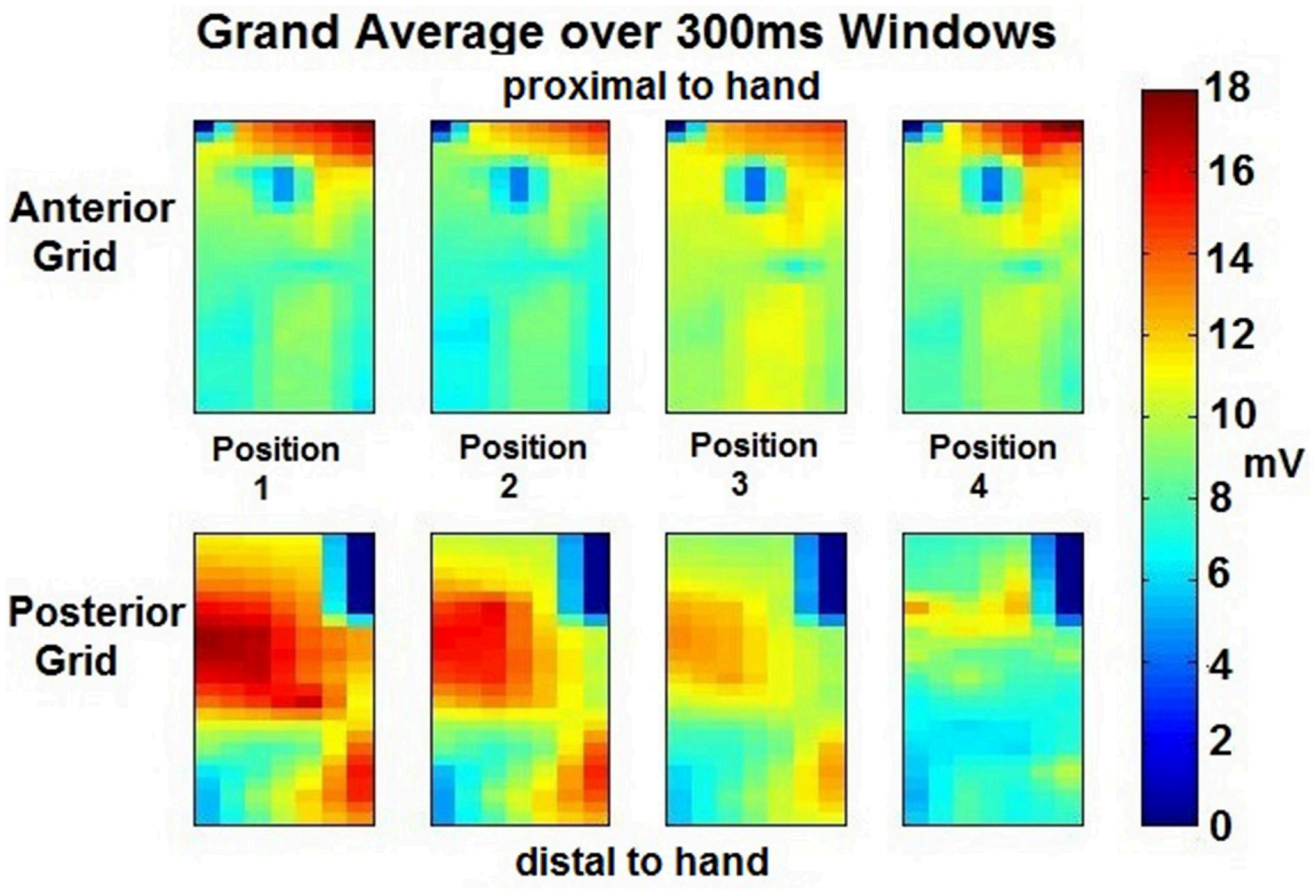
Grand Averaged Windows for Feature Extraction per position per ROI—The RMS values were obtained during 11 windows (300 ms each) of the isometric effort were averaged for each position of the thumb per grid placed on the forearm ROI. From the anterior grid (top 4 images), a different amplitude exertion is appreciated proximal to the hand, for the different thumb movements. A central exertion is appreciated which amplitude also varies depending on the position. An electrode at the center-top of these four images seems to have a clear detrimental in amplitude, this may be due to no activity on that area but most likely, and given the smooth transitions across the electrodes, it may be due to an electrode miss-contact. Similar analysis can be performed to the posterior grid (bottom 4 images). Clear amplitude difference is appreciated across positions, going from high amplitude to low amplitude from position 1 to 4. Position 4 seems to have different exertion and muscles involved than the rest of the positions, the amplitude is lessened but there are two sources of exertion at similar levels in amplitude and location.

RMS values for each isometric effort sustained were estimated using 300 ms window size with a 50 ms overlap for each electrode site in both ROI. The resulting windows were further averaged generating a highlighted spatial distribution map of each of the four opposition tasks. In Figure 4, an example of a participant's maps showing the presence of localized magnitude variations occurring in spite of consistency in grip force performance at each posture.

**Figure 5:**
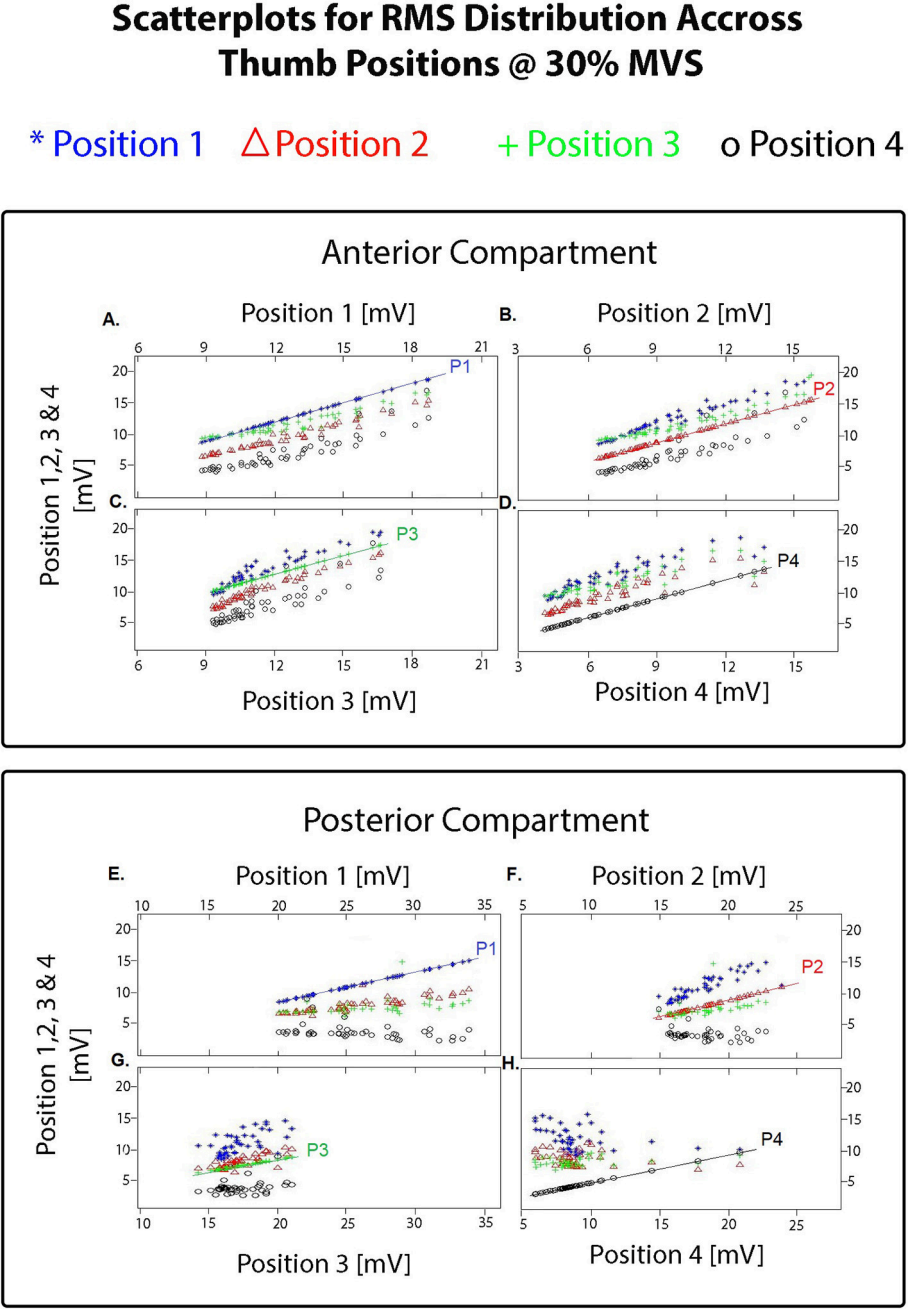
Resulting pair-wise scatterplots obtained using the Matlab Classification Learner toolbox (R2015a) on the averaged 300 ms overlapping windows of RMS vectors separated by thumb posture and by grid (totaling 7,680 vectors per grid). Each scatterplot shows the resulting clusters for each position as the comparator (i.e., P1 compared with P1 to 4; P2 compared with P1 to 4; and so on). For the electrode grid placed on the anterior compartment: **(A)** shows the resulting clusters highlighting the amplitude variation between position 1 of the thumb compared with position 1 (reference line on the graph labeled as P1), position 2, 3, and 4; **(B)** shows the resulting clusters highlighting the amplitude variation between position 2 of the thumb compared with position 2 (reference line on the graph labeled as P2), position 1, 3, and 4; **(C)** shows the resulting clusters highlighting the amplitude variation between position 3 of the thumb compared with position 3 (reference line on the graph labeled as P3), position 1,2, and 4; **(D)** shows the resulting clusters highlighting the amplitude variation between position 4 of the thumb compared with position 4 (reference line on the graph labeled as P1), position 1, 2, and 3. Similarly for the electrode grid placed on the posterior compartment with the posture comparison on **(E–H)**. As shown in these scatterplots and on the iso-potential maps on Figure 4, changes between the different thumb postures were only detected in amplitude.

A total of 1,920 vectors (64 electrodes × 30 repetitions; totaling 7,680 per grid) for each opposition and for each were then processed using the Classification Learner toolbox. The resulting clusters highlighting the amplitude variation across positions further separated by electrode grid position is shown in Figure 5. The anterior ROI displays a tight relation with a variability of 5 mV/mV. In contrast, on the posterior grid array, the thumb postures corresponding to position 1 to 3 share similar activation patterns with a greater variability (10 mV/mV), and position 4 displaying a tight spread between positions having a variability of <5 mV/mV and lower amplitude throughout (<10 mV/mV).

### Feature Classification Using SOFM and PCA

**Figure 6:**
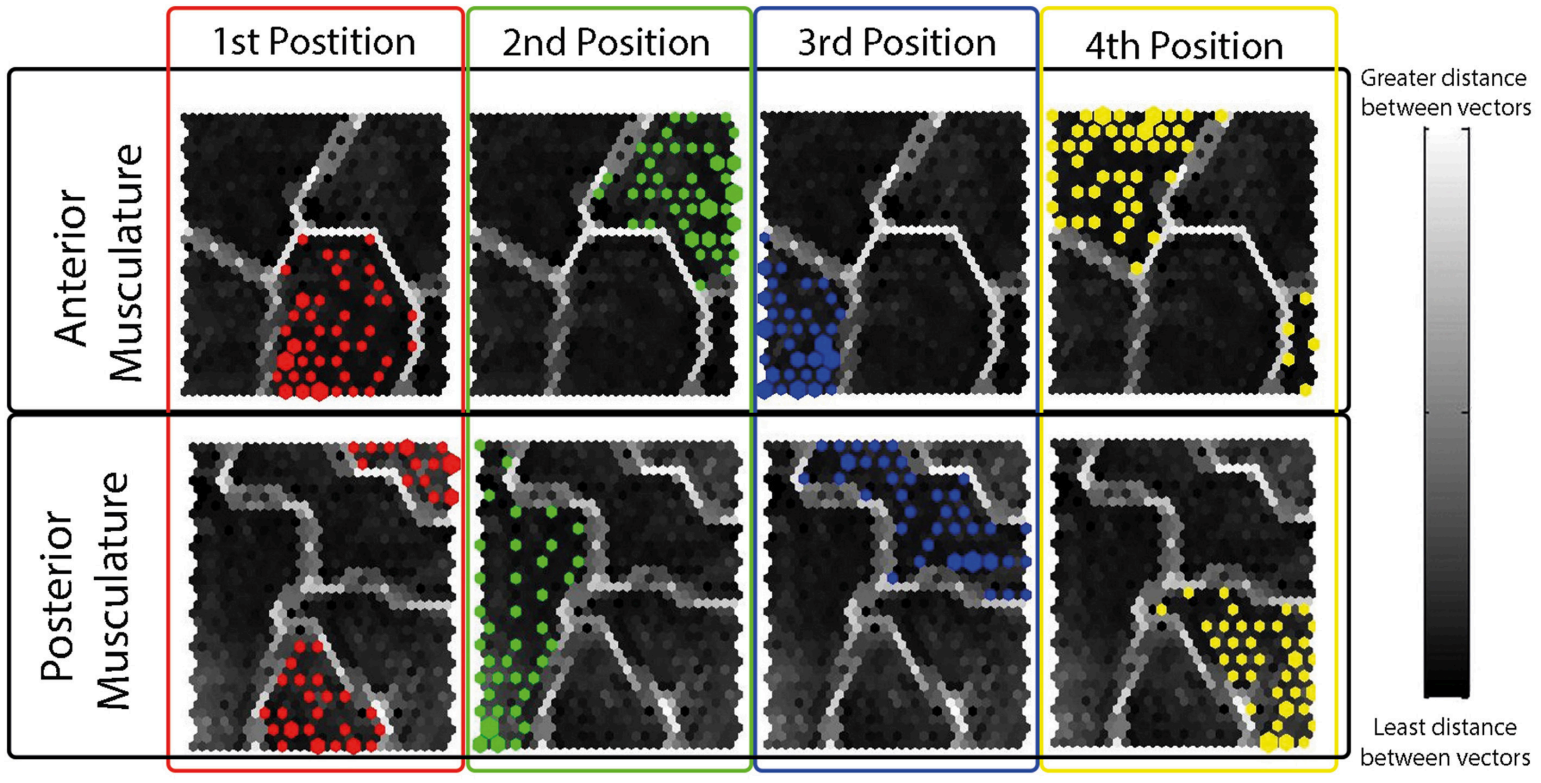
Resulting Hit Histograms from a Randomly Selected Subject during a Typical Test- Each hit histogram is shown on the resulting topographical map after the network was trained using the 60% of the data. The four different thumb grips have been separated and color coded by electrode grid locations (anterior musculature; posterior musculature). As observed, each position has a defined area in the topographical map different to the rest of the positions showing interrelations amongst same thumb opposition tasks.

The resulting 60% of the FVs containing the isometric effort of HD-sEMG data acquired from each participant was used to train the SOFMs. A resulting hit histogram, separated by thumb position, on the trained network during a test is shown in Figure 6.

The performance of the trained SOFMs was assessed through quantization, topographical and combined error as quality measures. The outcomes of these measures are shown in Table 1 for all the participating subjects. The errors measured were based on the vector projection (topology preservation) and vector quantization. These are shown in a scale from 0 to 100% where 100% means no significant difference between thumb opposition grips.

**Table 1:** Quantization, topographical and combined errors after training with SOFMs per subject per ROI (A: anterior musculature; P: posterior musculature).

**Subject**	**Quantization** **error (%)**		**Topographical** **error (%)**		**Combined** **error (%)**	
	***A***	***P***	***A***	***P***	***A***	***P***
1	1.04	1.08	<0.01	0.04	1.35	1.68
2	0.67	0.65	0.09	0.02	1.12	1.12
3	0.9	0.79	0.05	0.02	1.30	1.37
4	0.70	0.73	0.10	0.01	1.06	1.20
5	1.02	0.78	0.05	0.05	1.24	1.05
6	0.88	0.87	0.03	0.07	1.15	1.17
7	0.57	0.62	0.04	0.02	0.88	1.02

The remaining 40% of the data were used to test the SOFMs to compare the results of the un-presented datasets to the already trained network. The precision and success is shown on Table 2.

**Table 2:** Performance measures for SOFMs test data—Separated by forearm flexor (A) and extensor muscles (P) for each thumb grip formation (1st, 2nd, 3rd, and 4th).

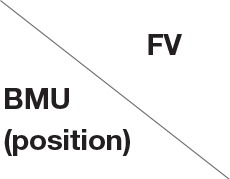	**1st position** **(%)**		**2nd position** **(%)**		**3rd position** **(%)**		**4th position** **(%)**	
	***A***	***P***	***A***	***P***	***A***	***P***	***A***	***P***
1st	87	91	4	6	5	0	4	3
2nd	7	5	83	82	3	6	7	7
3rd	0	3	8	5	86	88	6	4
4th	6	1	5	7	6	6	83	86

*Red corresponds to position 1; Blue corresponds to position 2; Green corresponds to position 3; Yellow corresponds to position 4*.

**Figure 7:**
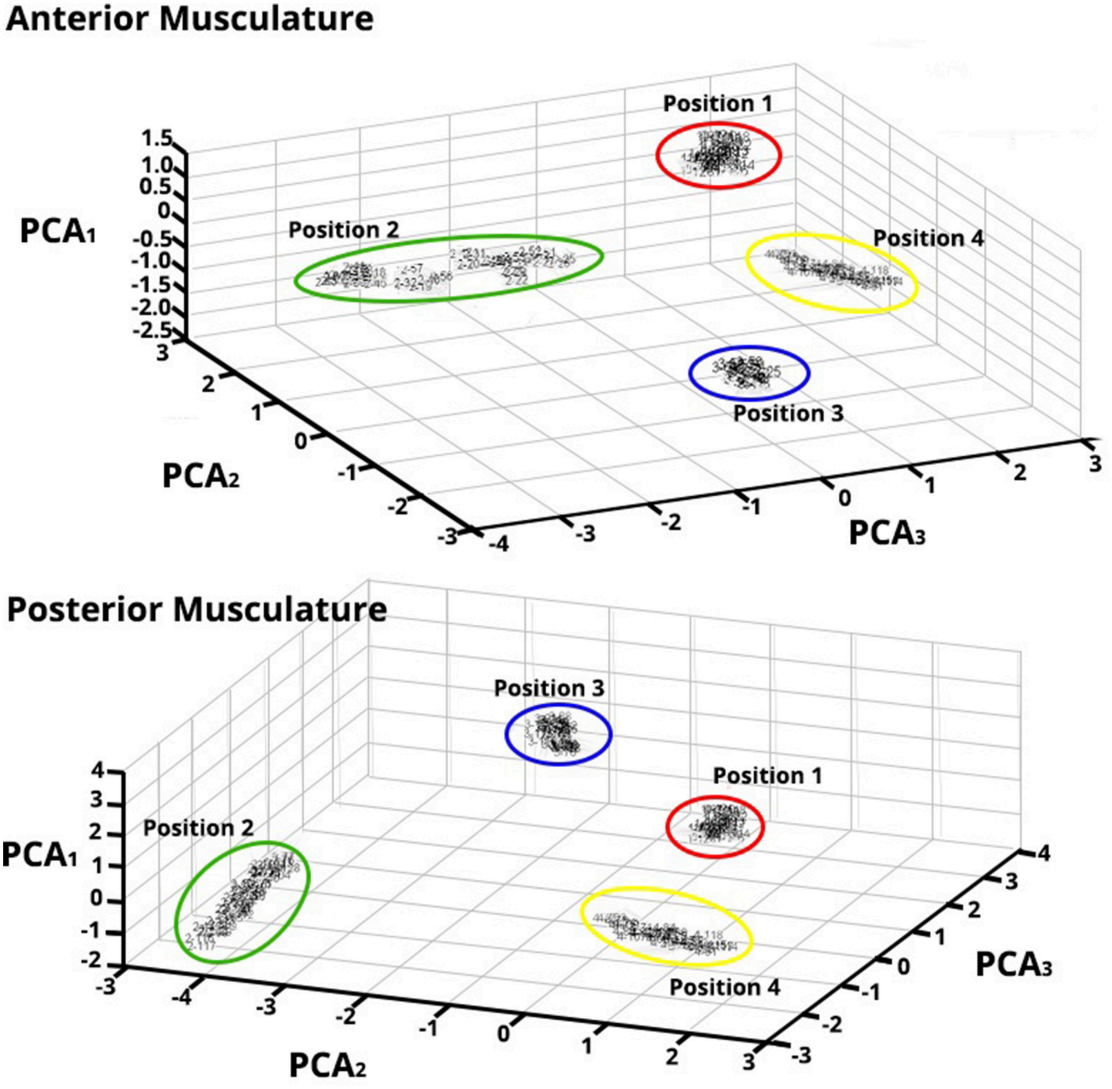
Principal Component Analysis (PCA) for Feature Vectors (FV) after Data Were Trained applying SOFM—Forearm Flexor Muscles **(top)** and Extensor Muscles **(bottom)**—PCA of the FV after SOFMs were applied to the trained data for a randomly chosen subject. As depicted, each color represents each position that the thumb was opposed to. The resulting PC conversion shows tight clusters; this clustering also reflects the wide distance between neighborhoods. Note that the PCA1 axes are of different amplitude highlighting the different magnitude responses in each ROIs.

The different thumb positions were further inspected reducing the dimensions through PCA (Figure 7). This analysis was performed using the maps trained by the SOFMs in order to visualize the clustering areas in the new PCs. The results are shown in Figure 6 and highlight the tight clustering formation between same grip patterns but a wide spread across different grip patterns.

This accentuates that important, classifiable differences are found in the HD-sEMG patterns extracted from the extrinsic hand muscles while the hand performs different grips.

### Data Validation Through Supervised Neural Networks

Supervised neural networks (NN), such as LM and the NNPR were applied using the MATLAB Pattern Recognition toolbox for further data validation. The HD-sEMG data inputs were provided with the appropriate labels so that the network could recognize which cluster each of the presented FV belonged to. The NN performance was assessed through the values of cross-entropy and percentage of error (Table 3), and confusion matrices (Table 4), as these are common performance measures used in these NN methodologies.

**Table 3:** Parameter distribution and the resulting cross-entropy and percentage error measures from supervised Levenberg-Marquardt validation (Matlab routine) on the two ROIs: Anterior and posterior musculature.

		**Anterior**			**Posterior**		
**Subject**	**Stage**	**Samples**	**Cross-entropy**	**% Error**	**Samples**	**Cross-entropy**	**% Error**
1	Training	1,476	6.23	<0.01	1,368	2.85	0.21
	Validation	1,722	5.82	0.05	1,596	2.70	3.94
	Testing	1,722	8.82	0.11	1,596	2.71	6.20
2	Training	1,656	9.27	<0.01	1,368	2.85	0.21
	Validation	1,932	8.62	<0.01	1,596	2.70	3.94
	Testing	1,932	8.62	0.05	1,596	2.71	6.20
3	Training	1,908	1.90	17.24	1,728	3.29	2.08
	Validation	2,226	1.81	19	2,016	3.10	4.31
	Testing	2,226	1.80	16.03	2,016	3.12	5.20
4	Training	1,908	5.67	<0.01	1,620	4.68	0.06
	Validation	2,226	5.29	0.22	1,890	4.36	0.74
	Testing	2,226	5.26	0.17	1,890	4.36	1.26
5	Training	2,052	5.40	0.34	1,260	4.23	<0.01
	Validation	2,394	5.06	1.29	1,470	3.94	0.06
	Testing	2,394	5.10	1.37	1,470	3.93	<0.01
6	Training	1,656	5.72	<0.01	1,728	5.05	<0.01
	Validation	1,932	5.32	0.05	2,016	4.70	0.44
	Testing	1,932	5.33	0.31	2,016	4.70	0.24
7	Training	1,260	4.46	17.85	1,584	4.21	6.88
	Validation	1,470	4.20	18.02	1,848	3.93	9.36
	Testing	1,470	4.19	17.89	1,848	3.94	7.94

**Table 4:** Accuracy of the supervised classification methods for the flexor and extensor muscles by stages of neural network: training, validation and testing.

	**Anterior musculature of the forearm**						**Posterior musculature of the forearm**					
**Subject**	**Accuracy (%)**			**Misclassification (%)**			**Accuracy (%)**			**Misclassification (%)**		
	**Train**	**Val**.	**Test**	**Train**	**Val**.	**Test**	**Train**	**Val**.	**Test**	**Train**	**Val**.	**Test**
1	100	99.9	99.9	0.0	0.1	0.1	99.8	96.1	93.8	0.2	3.9	6.2
2	100	100	99.9	0.0	0.0	0.1	100	100	99.9	0.0	0.0	0.1
3	82.8	81.8	84.0	17.2	19.0	16.0	97.9	95.7	94.8	2.1	4.3	5.2
4	100	99.8	99.8	0.0	0.2	0.2	99.9	99.3	98.7	0.1	0.7	1.3
5	99.7	98.7	98.6	0.3	1.3	1.4	100	99.9	100	0.0	0.1	0.0
6	100	99.9	99.7	0.0	0.1	0.3	100	99.6	99.8	0.0	0.4	0.2
7	82.1	82.0	82.1	17.9	18.0	17.9	93.1	90.6	92.0	6.9	9.4	8.0
Total	(94.85 ± 8.10)			(5.14 ± 8.10)			(97.00 ± 3.37)			(3.00 ± 3.31)		

It can be observed from the Table 4, that the classification accuracy achieved across the anterior array across all participants was 94.85 ± 8.10% with a misclassification of 5.14 ± 8.10%. Performance from posterior array classification was slightly enhanced in comparison with an accuracy of 97 ± 3.37% and a misclassification of only 3 ± 3.31%. These higher rates of classification accuracy across ROIs highlight the complex functional anatomy and necessary synergistic activation that exists between forearm and intrinsic hand muscles during even simple thumb grip formations.

## Discussion

It remains a technical and economic challenge to provide adequate and consistent user control of powered hand prosthesis from the small numbers of sEMG electrodes implemented in current devices with limited processing power and running simple signal processing protocols. As the complexity and functional capability of next generation powered anthropomorphic hands increases, so does the need for better and more intuitive user-control. Presently, no myoelectric prosthesis provides sEMG based control over thumb components. This is surprising from an anatomical, physiological and functional perspective as the thumb is the digit which gives the human hand its greatest adaptability in grip and gesture formation, and it is the digit most often coupled to the motions of the other digits in grip and object manipulation. The thumb is estimated to be contribute to at least 50% of normal overall hand function (Park et al., 2012).

As coordinated motions of the digits are driven by user intention executed through muscle synergies there must be embedded within the EMG activation patterns a predictable outcome of what actions an individual's hand is being commanded to perform. Within this pattern will also be information relating to the use of the thumb in gesture and grip actions. The proposition to control prosthetic hands by identifying muscle synergies or patterns is not new (e.g., see Castellini and van der Smagt, 2013), but there has been very little research investigating if sEMG pattern recognition approaches have the potential to extract information on intended thumb actions as part of grip formation. The preliminary aim of this study was to investigate if information on differing thumb actions can be recovered from the sEMG patterns sampled from the forearm musculature. We are interested in these muscle groups as opposed to the intrinsic hand muscles as these are the muscle groups that survive hand amputation and remain under voluntary control. The forearm musculature is largely associated with control of digit extension/flexion (digits 2, 3, 4, and 5) and actions of the wrist. The four extrinsic muscles of the thumb (FPL, EPL, APL, and EPB) in contrast to the other wrist and hand muscles have relatively low muscle mass and are located in deep forearm compartments resulting in a relatively low and filtered contribution to standard sEMG recordings. Only FPL and APL have a functional action in generating thumb tip force during grip closure. This study was therefore designed as a proof of concept investigation and serves as a precursor to further work on amputees and on the development of alternative methods of providing myoelectric control over anthropomorphic prosthetics.

### Amplitude Signal Analysis

Preliminary visualization and RMS analysis strongly implies that classifiable differences between muscle activation patterns during contractions associated with the performance of the four different thumb opposition postures can be resolved. These differences result from an amplitude modulation and not changes in the location of activity. The significance of this result is that sEMG recordings dominated by activity from forearm muscles that have no direct action on the thumb, display patterns of activation that co-vary in relation to thumb position.

Common to all subjects was the observation of higher signal amplitudes in the proximally clustered electrode recording sites from the posterior forearm and relatively low levels of sEMG activity from electrodes in the distal portions of the anterior or posterior forearm. This suggests a more complex co-activation of anterior and posterior muscle groups exist in the performance of the grip tasks requested of volunteers that would be anticipated through a simple consideration of the anatomical actions of the muscle groups recorded from. The use of HD-sEMG in these experiments provides a highly effective way of visualizing muscle synergies and aids in identifying the key recording sites where pattern recognition may achieve the highest yields even in muscles where there is no direct action on positioning the thumb or contributing to the generation of thumb tip force. Importantly, the use of HD-sEMG allows for optimization of electrode number and locations when designing real-time applications where computational cost and number of recording sites are important considerations.

### Machine Learning Analysis

The use of machine learning and pattern recognition algorithms has been applied in upper limb prosthetics in many studies (e.g., Hiraiwa et al., 1989; Yang et al., 2009; Arjunan et al., 2010; Varol et al., 2010; Edwards et al., 2016; Gailey et al., 2017). Algorithms with high classification accuracy and potential for use in prosthetic devices include (but are not limited to): multilayer perception (MPL) (Nielsen et al., 2009; Muceli and Farina, 2012); linear discriminant analysis classifier (LDA) (Yonghong et al., 2005; Phinyomark et al., 2012; Celadon et al., 2016); common special patterns proportional estimator (CSP-PE); and a thresholding (THR) algorithm (Celadon et al., 2016).

Unsupervised learning are effective tools to use when categorizing and clustering different inputs. Based on the results presented, these methods correctly distinguished between the different positions of the thumb based on the activity patterns recorded. This reaffirms the utility of basing the analysis on high density sampling of recording sites in order to gain representation of the changes that occur in the muscle activity profiles associated with each thumb opposition task. Improved accuracy is likely to be achievable when measures of co-variation across compartments and electrode sites are considered and recording configurations for prosthetic control purpose can be optimized to achieve the minimal numbers of electrodes necessary for adequate control.

In this research, the overall performance that SOFMs achieved when assessing anterior musculature was 88.25 ± 0.5% and for the posterior musculature overall performance was 91.25 ± 2.5%. These values are favorable with respect to previous published data on grip/gesture classification rates and the current machine learning SOFMs performance is acceptable for use in offline device control. However, even with this accuracy the error rate would be unacceptable in a commercial device and further research has to be conducted to further understand muscle synergies during different hand movements.

An important practical extension of this work is the need to look at the dynamic phases of force development during different grip transitions and the modulation of grip force itself and to recreate the paradigm tested in amputee subjects.

In relation to previous research the authors believe that the work presented provides an important demonstration that indicators of variations in thumb position can be derived from a consideration of activity dominated by the EMG from extrinsic hand and forearm muscles.

Critically, as mentioned above this study only gathered data for a single grip force level and did not investigate the dynamic grip transitions or the change in the position of the thumb from one posture to another. Further work in this context is necessary before real-time active myoelectric control over a prosthetic thumb component in a device can be considered for transradial amputees.

## Data Availability

The raw data supporting the conclusions of this manuscript will be made available by the authors, without undue reservation, to any qualified researcher.

## Ethics Statement

This study was approved by the University of Strathclyde Research Ethics Committee and in accordance with the Declaration of Helsinki). All subjects provided informed written consent prior to the tests.

## Author Contributions

AA-G and BC jointly planned the research protocol. AA-G carried out the testing, signal acquisition and analysis. AA-G and BC jointly analyzed and discussed the findings. Both contributed to the production of the manuscript.

### Conflict of Interest Statement

The authors declare that the research was conducted in the absence of any commercial or financial relationships that could be construed as a potential conflict of interest.
